# Obesity, dysbiosis and inflammation: interactions that modulate the efficacy of immunotherapy

**DOI:** 10.3389/fimmu.2024.1444589

**Published:** 2024-08-26

**Authors:** Ashutosh S. Yende, Dipali Sharma

**Affiliations:** Department of Oncology, Johns Hopkins University School of Medicine and Sidney Kimmel Comprehensive Cancer Center at Johns Hopkins, Baltimore, MD, United States

**Keywords:** obesity, gut microbiota, breast cancer, dysbiosis, immunotherapy, inflammation

## Abstract

Recent years have seen an outstanding growth in the understanding of connections between diet-induced obesity, dysbiosis and alterations in the tumor microenvironment. Now we appreciate that gut dysbiosis can exert important effects in distant target tissues via specific microbes and metabolites. Multiple studies have examined how diet-induced obese state is associated with gut dysbiosis and how gut microbes direct various physiological processes that help maintain obese state in a bidirectional crosstalk. Another tightly linked factor is sustained low grade inflammation in tumor microenvironment that is modulated by both obese state and dysbiosis, and influences tumor growth as well as response to immunotherapy. Our review brings together these important aspects and explores their connections. In this review, we discuss how obese state modulates various components of the breast tumor microenvironment and gut microbiota to achieve sustained low-grade inflammation. We explore the crosstalk between different components of tumor microenvironment and microbes, and how they might modulate the response to immunotherapy. Discussing studies from multiple tumor types, we delve to find common microbial characteristics that may positively or negatively influence immunotherapy efficacy in breast cancer and may guide future studies.

## 1 Introduction

The obesity pandemic currently affects over 42% of the United States population, and a staggering one billion people worldwide are living with obesity (1). The past four decades have seen obesity progress from an epidemic to a global pandemic. This is further evident from the steady increase in mean Body Mass Index (BMI), a measure of obesity, across the globe. For instance, the global mean BMI, tracked between 1975 and 2014, swelled from 21.7 kg/m^2^ to 24.2 kg/m^2^ in men, and from 22.1 kg/m^2^ to 24.4 kg/m^2^ in women, respectively (2). During this period, the number of obese individuals, as defined by a BMI of ≥30 kg/m^2^, has more than tripled. Obesity, which is typically marked by an increase in adiposity, has been linked with a plethora of pathological conditions such as hypertension, type 2 diabetes, cardiovascular diseases, musculoskeletal and kidney disorders, and even cancer (3, 4). The International Agency for Research on Cancer now recognizes thirteen types of cancers that are poorly affected with obesity: namely, esophageal cancer, stomach cancer, pancreatic cancer, gallbladder cancer, liver cancer, postmenopausal breast cancer, uterine cancer, ovarian cancer, meningioma, thyroid cancer, kidney cancer, colorectal cancer and multiple myeloma (5). Some of the factors contributing to the negative prognosis in obese patients include the tumor promoting adipocytokine-rich microenvironment, increased levels of growth factors and inflammatory cytokines which fuel cancer progression and associate with poor response to therapy (6, 7). It is well-established that weight gain in late adulthood and after menopause significantly increases the risk of developing breast cancer (8, 9).

Obese state is also associated with gut dysbiosis. The gut microbiota represents trillions of microorganisms that live as commensals. These include diverse species of bacteria, archaea, fungi and viruses which secrete discrete metabolites that affect vital physiological processes in the gut, as well as distant organs (10, 11). Composition of the gut flora varies greatly between individuals and is often determined by host lifestyle and eating habits, genetic factors, ongoing disease conditions and treatments as well as antibiotics usage (11, 12). Microbes form a micro-ecosystem within the host and maintain a state of physiological homeostasis (10). The resident microbiota is critically involved in maintaining host defenses against pathogens and pathobionts (13). Additionally, the gut microbiota also promotes metabolic events in the gut to generate energy by facilitating degradation of otherwise indigestible carbohydrates and proteins through microbial enzymes (14, 15). Alterations in the gut microbiota have been linked to obesity, diabetes, gastrointestinal disease as well as cancer (10, 16–21). In fact, an ever-increasing body of evidence suggests the gut microbiota to be a major player/variable in the obesity-cancer axis (22, 23). Several gut microbial species, such as *Akkermansia*, *Clostridiales*, *Ruminococcaceae*, and *Faecalibacterium*, have been liked to improved response to cancer therapy (24–27). Mechanistically, it has been suggested that metabolites secreted by the diverse gut microbial flora influence the breast tumor microenvironment (TME) (28, 29), and act on immune signaling cascades thereby modulating immune responses within the TME (24, 30, 31). Dysbiosis in the gut ecosystem, common in obese breast cancer patients, affects the metabolic composition around the breast tissue which consequently influences tumor progression, response to therapy, and disease outcome (29, 32). In this review, we summarize recent progress in the connections between breast cancer, obesity and microbiota, to understand the interplay, and identify potential areas of interest for future research.

## 2 Obesity negatively impacts breast cancer

Breast cancer is the most frequently diagnosed cancer and the leading cause of cancer-related deaths among women in the United States. It is estimated that, in 2024 alone, about 310,720 women and 2,790 men in US will be diagnosed with breast cancer (33, 34). Apart from the genetic, gender and age-related risk factors, several modifiable risk factors such as eating habits, lack of physical exercise, obesity, smoking and alcohol-drinking are associated with breast cancer incidence. The relationship between obesity and breast cancer is of particular interest as premenopausal women who are either overweight or obese, show reduced occurrence of breast cancer (35), however, in postmenopausal women, increased incidences of breast cancer are strongly correlated with higher BMI (36–38). Obese breast cancer patients with BMI ≥35 kg/m^2^, in particular postmenopausal women, demonstrate significantly higher hazard ratios for the development of hormone receptor positive (ER+/PR+) breast cancer, with larger tumors and more advanced disease (37, 39). An alarming 12% increase in breast cancer risk with every 5 kg/m^2^ BMI increase in overweight and obese postmenopausal women was revealed through a meta-analysis (40). The Women’s Health Initiative clinical trial which enrolled ~67,000 postmenopausal breast cancer patients aged 50-79 years, also reported a greater risk of developing invasive breast cancer in overweight and obese women (39). The TME in obese patients is dominated by excess adipocytes surrounding the tumor. Mechanistically, estrogens derived from adipose tissue by increased aromatization of androstenedione substitute for the lack of ovary-derived estrogens in post-menopausal stage and fuel the hormone receptor (ER+PR+) positive tumors (41–43). Obese and overweight state in breast cancer patients present additional set of challenges ranging from poorer initial screening, presence of comorbidities, complications in surgery to reduced efficacy of chemotherapeutic drugs and endocrine therapy (44–46). Some studies have suggested higher drug toxicities in obese breast cancer patients which may lead to dose de-escalation and poor overall survival (47, 48). Although the relationship between obesity and endocrine therapy resistance in breast cancer has been inconclusive in clinical studies (46, 49–53), our preclinical studies uncovered the molecular mechanisms by which obesity might mediate endocrine therapy resistance (54, 55).

## 3 Obese state yields major alterations in breast tumor microenvironment

As with any malignancy, the breast TME greatly influences the tumor growth and progression. The breast TME comprises of stromal cells such as fibroblasts, adipocytes, endothelial and immune cells, as well as soluble factors (55, 56), and tumor cells are known to modify the stromal cells facilitating the expression of genes necessary for tumor growth and stemness (55, 57). Cancer-associated adipocytes (CAAs) within the stroma, secrete chemokines, adipokines, growth factors, extracellular matrix (ECM) modifying enzymes and proteins that promote tumor growth and metastasis (57, 58). Cancer associated fibroblasts (CAFs) which form majority of the tumor stroma, secrete growth factors such as vascular endothelial growth factor (VEGF) and transforming growth factor β (TGFβ) that further promote angiogenesis and fibrosis within the TME (55) and help tumor growth.

### 3.1 Modulation of adipokines, steroid hormones and growth factors mediates the molecular effects of obesity

Obesity brings about endocrine and metabolic reprogramming which promotes tumor growth. Adipose tissue is the source for key adipokines such as adiponectin and leptin and the ratio of adiponectin/leptin is adversely affected in obese breast cancer patients. While level of adiponectin which exerts protective effects against obesity-related breast cancer progression is reduced, expression of leptin is strongly increased in obese state (59, 60). Elevated leptin level (hyperleptinemia) not only promotes obesity-associated inflammation, but also potentiates tumor growth, invasion and metastasis (55, 60). Hyperleptinemia also interferes with tamoxifen efficacy in luminal breast cancer (55). Moreover, excess adipose tissue contributes to hormonal dysregulation. In obese women, particularly after menopause, estrogens are derived almost exclusively from adipose tissue, and adipocytes demonstrate higher activity of aromatase, an enzyme which is critical to estrogen biosynthesis (61). Furthermore, the inflammatory signaling in the TME, and the crosstalk between macrophages and stromal cells is also suggested to promote aromatase activity (60). Thus, estrogen-rich adipose environment promotes the growth of ER+ breast tumors. The relationship between obesity and risk of recurrence in breast cancer patients treated with adjuvant aromatase inhibitor has been further highlighted in a recent study conducted on a cohort of 13,230 patients with ER+PR+ breast cancer. Essentially, high BMI associated with increased risk of breast cancer recurrence in patients treated with aromatase inhibitor (62). These results support the findings of Arimidex, Tamoxifen Alone or in Combination (ATAC) trial which showed that women in higher quintile of obesity present poorer prognosis as compared to lean women (63). Additionally, increased levels of circulating insulin and insulin-like growth factor 1 (IGF1), typically associated with high BMI and obesity, positively influence aromatase activity in the adipose tissue. Hyperinsulinemia is reported to not only increase the risk of developing post-menopausal breast cancer but also cancer recurrence and mortality (61, 64). Exhibiting a bidirectional crosstalk, tumor cells restructure endocrine and metabolic signaling in surrounding adipocytes to promote tumor growth, stemness and endocrine therapy resistance (55, 60, 61). Ambrosio et al. reported an increase in connective tissue growth factor (CTGF) mRNA with reduced sensitivity to tamoxifen in breast cancer cells cocultured with mammary adipocytes under conditions of high glucose, an effect that was reversed by inhibiting adipocyte-derived interleukin 8 (IL8) (65). In another study, sera obtained from obese breast cancer patients was demonstrated to promote viability, growth and endocrine resistance of breast cancer cells via ERα-mediated activation of the PI3K/Akt signaling pathways (66). Additionally, using a high fat diet (HFD)-induced obese murine model to develop ER+ patient-derived xenografts (PDXs), Wellberg et al. showed that obesity and excess energy form a TME that is conducive to endocrine resistance, and also identified fibroblast growth factor receptor 1 (FGFR1) signaling as a critical mediator (67). Along similar lines, mice fed with a fasting mimicking diet (FMD) displayed superior tumor-inhibition in response to tamoxifen and fulvestrant compared to control diet fed mice (68). Further analysis revealed that FMD mice had lower circulating levels of IGF1, insulin and leptin along with upregulation of EGR1 and PTEN which inhibited the AKT-mTOR signaling axis. Moreover, fulvestrant and cyclin-dependent kinase (CDK) 4/6 inhibitor palbociclib, when combined with FMD, promoted superior tumor regression and also reversed acquired resistance (68). Overall, obesity-associated endocrine deregulation marked by alterations in adiponectin, leptin, insulin, IGF1 and estrogens contributes to increased risk of cancer progression and recurrence in obese breast cancer patients. These studies warrant consideration of BMI in therapeutic decisions, and including higher doses of tamoxifen or aromatase inhibitors as well as improved combination regimens that can effectively treat obese ER+PR+ breast cancer patients.

### 3.2 Obese state fosters a hypoxic tumor microenvironment to support breast cancer growth

Adipocyte hypertrophy and hyperplasia are commonly observed in obese state (69). In breast TME, a vast number of enlarged adipocytes with large lipid molecules surround the tumor, that eventually lowers oxygen availability and increases oxidative stress (69, 70). Thus, adiposity changes the TME landscape by creating a hypoxic and inflammatory environment that is rich in cytokines and free fatty acids (55, 71). Hypoxia, in turn causes endoplasmic reticulum stress that manifests into dysregulated adiponectin secretion by adipocytes (72). These changes further reduce the metabolic flexibility of adipocytes, thereby increasing the rate of apoptosis and ultimately accumulating more inflammatory cells around the tumor. Hypoxia triggers the release of excessive chemokines and inflammatory cytokines that promote the recruitment of tumor associated macrophages and escape from immune surveillance (73, 74). Using 4T1 murine breast cancer mouse models subjected to 8% O_2_ for 6h/day to create the hypoxic environment, Wang et al. showed that hypoxia promotes galectin-3 expression and macrophage infiltration in tumors to promote angiogenesis and metastasis (73). Hypoxic conditions trigger the activation of hypoxia-induced factor 1 (HIF-1) in adipocytes which is known to initiate metastasis and adversely affect patient survival (75, 76). FVB mice harboring *Hif-1α* knockout mammary tumor epithelial cells displayed reduced tumor growth, reduced lung metastasis and increased survival compared to wild type cells (77). Several studies suggest that the hypoxic environment and oxidative stress within obese TME also impact treatment efficacy (53, 78, 79). In fact, stable HIF-1α overexpression in MCF7 cells resulted in loss of sensitivity to the selective ER degrader fulvestrant, while its knockdown sensitized the cells to fulvestrant (78). Inhibition of HIF-1α transcription with zoledronic acid, a drug used to treat postmenopausal osteoporosis, proved useful to potentiate endocrine therapy. Further, MCF7 cells subjected to chronic oxidative stress through exposure to hydrogen peroxide readily transformed to become estrogen-independent with aggressive growth characteristics (80). Increased phosphorylation of Jun NH (2)-terminal kinase (JNK) and c-Jun, and elevated AP-1 activity were suggested to be one of the mechanisms involved in transformation of MCF7 xenografts from tamoxifen sensitive to tamoxifen resistant (79). An improved understanding of hypoxia and its molecular impact within the breast TME can potentially lead to the development of promising targets for overcoming drug resistance and enhancing the efficacy of current therapeutic regimens. While many direct inhibitors of HIF-1α have shown promising results in preclinical studies (74, 81), comprehensive breast cancer clinical trials are still needed.

### 3.3 Sustained low grade inflammation is a characteristic feature of tumor microenvironment in obese state

**Figure 1:**
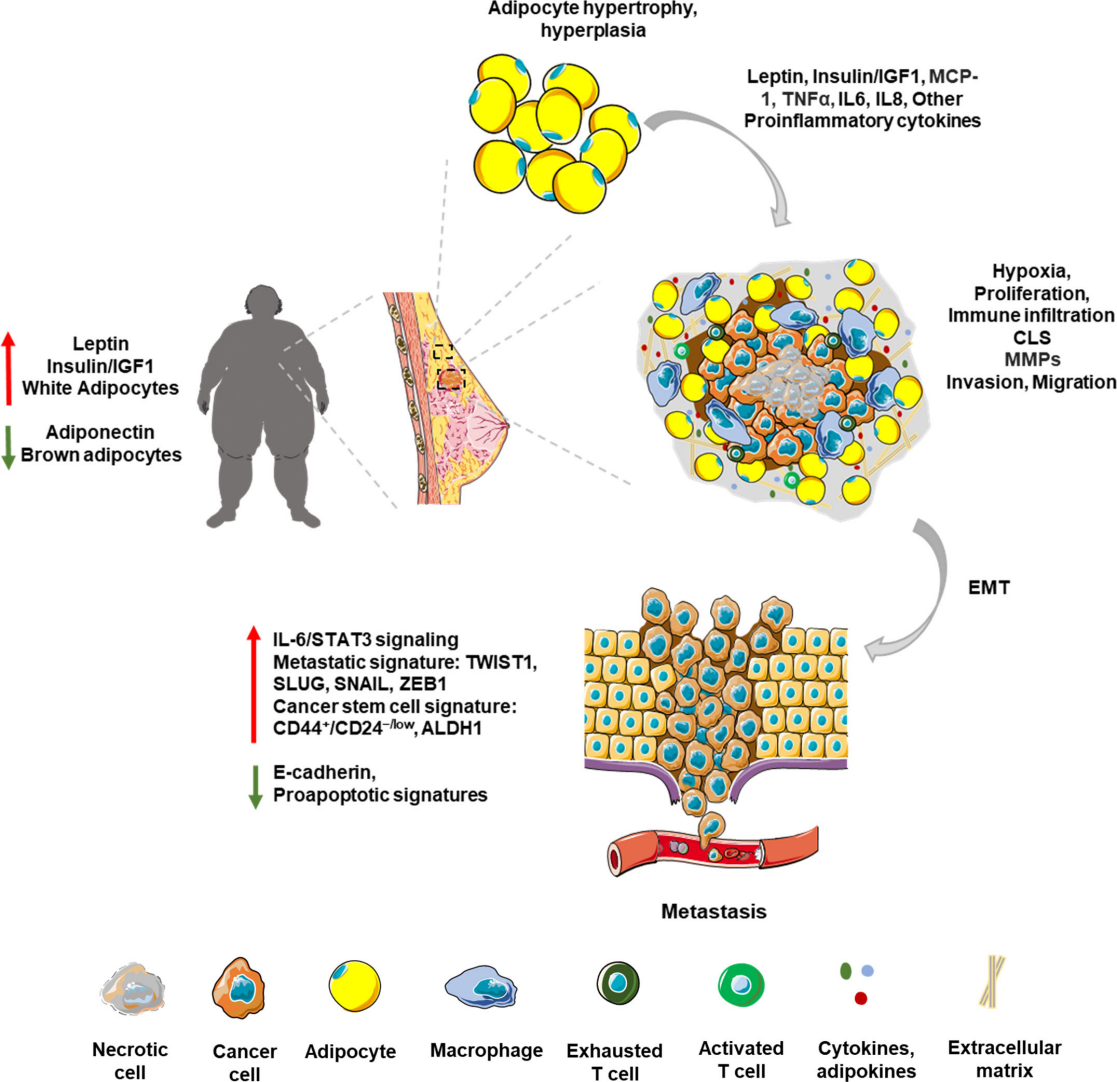
Obese breast tumor microenvironment: The breast TME is a complex network influenced by various cell types and secreted factors, crucial for tumor growth and progression. Stromal cells like fibroblasts and adipocytes, and immune cells like macrophages and T cells, play pivotal roles in shaping this environment. Obesity-associated breast TME is marked by adipocyte hypertrophy and hyperplasia. Adiposity exacerbates tumor growth through endocrine and metabolic reprogramming, altering adipokines such as leptin and adiponectin. High adiposity leads to hypoxia within the TME, fostering a pro-inflammatory milieu rich in cytokines. Chronic low-grade inflammation associated with obesity attracts macrophages and other immune cells, forming focal inflammatory points and crown-like structures (CLS). This inflammatory microenvironment is marked by the presence of proinflammatory molecules like TNFα and IL6. Tumor cells manipulate stromal architecture by producing matrix metalloproteinases (MMPs) leading to invasion and migration of cancer cells. This results in epithelial to mesenchymal transition (EMT) of cancer cells leading to increased expression of metastatic and stem cell signatures.

Obesity, in general, marks a state of chronic low-grade inflammation which provides supportive TME for the tumor cells, promoting mutational and epigenetic changes that favor growth and metastasis (82). Adipocyte hypertrophy, hyperplasia, hypoxia and the resultant inflammation lead to infiltration by macrophages (37, 57, 60). These macrophages in turn release proinflammatory signals such as tumor necrosis factor α (TNFα), cyclooxygenase-2 (COX-2), IL6, IL1β and monocyte chemoattractant protein-1 (MCP1) (60). TNFα has been found to play complex roles in cancer. While it has anticancer properties primarily through inducing cancer cell death—a process that can be harnessed for cancer therapy—it can also promote tumor growth in many cancer cells resistant to TNF-induced cytotoxicity by stimulating proliferation, survival, migration, and angiogenesis. In obesity-associated tumors, the pro-inflammatory and tumor-promoting effects of TNFα seem to be more prominent (83, 84). MCP1 stimulates the recruitment of monocytes which polarize to proinflammatory M1 macrophages in adipose tissue. Of note, adipose tissue from obese individuals displays predominantly M1 macrophages while M2 macrophages are more common in adipose tissue from lean individuals (85). The proinflammatory M1 macrophages accumulate around the dying adipocytes, forming focal inflammatory points referred to as crown like structures (CLS), which in turn have been positively correlated with higher BMI (86–89). In addition to necrotic adipocytes and proinflammatory macrophage signature, CLS are also marked by an increased infiltration of other immune cells including CD8+, Th1 and Th17 T lymphocytes, neutrophils and mast cells (90–92). In fact, CD8+ T cell infiltration is suggested to precede macrophage recruitment to CLS (91). Moreover, Interferon‐gamma (IFN-γ) produced by Th1 and CD8+ T cells stimulates polarization of macrophages to M1 (91, 93). Adipocytes also produce excessive levels of granulocyte-macrophage colony stimulating factor (GM-CSF), which induces recruitment and differentiation of neutrophils (94). Following this, immunosuppressive cell types such as T-regulatory cells and myeloid derived suppressor cells (MDSCs) are recruited at the inflammation site (94). Chronic low-grade inflammation in obesity also leads to the activation the proinflammatory oncoprotein nuclear factor κB (NF‐κB) (95), which is a constitutively active, well-established negative prognostic factor for breast cancer as it promotes tumor progression, invasion, stemness and also, endocrine resistance (96–102). Enhanced NF‐κB activity negatively impacts tamoxifen efficacy in ER+ breast cancer cells, and inhibition of NF‐κB signaling rendered the cells sensitive to tamoxifen (103–105). Combining NF‐κB-targeting proteasome inhibitor with endocrine therapy provides improved outcomes in patients with therapy-resistance and aggressive metastatic disease (106, 107). In addition, several inflammatory mediators have also been shown to play a role in conferring endocrine resistance. IL6, for instance, acts via STAT3-signaling pathway to regulate self-renewal of hormonal therapy-resistant cancer stem cells (108, 109). More recently, the cytokine CC‐chemokine ligand 2 (CCL2) produced by tumor associated macrophages was shown to induce tamoxifen resistance in MCF7 cells via activation of the PI3K/Akt/mTOR axis (104). Obese state, by virtue of modulating the immune TME impacts tumor progression as well as response to therapy and recurrence. **Figure 1** provides a summary (**Figure 1**). A recent study conducted on a Chinese cohort proposed four biomarkers—adiponectin, soluble leptin receptor, resistin, and C-reactive protein—as indicators of increased breast cancer risk in obese postmenopausal women (110). It is essential to methodically analyze these markers across a broader population and assess their relevance in predicting therapy resistance. Obesity contributes to the growth and metastatic dissemination of breast cancer cells in multiple ways. Key features of obesity-associated breast cancer including endocrine deregulation, hypoxia, and chronic low-grade inflammation are associated with complex molecular signaling networks, and several of these molecular nodes are currently being explored as predictive biomarkers for recurrence and therapy resistance.

## 4 Obese state and gut microbial dysbiosis form a close partnership that fuels breast cancer

Gut microbial dysbiosis alters metabolic processes leading to eating disorders and weight gain, through modification of the gut-brain axis (111, 112). Metagenomic analysis of obese vs lean gut microbiota revealed differences in relative abundance of two predominant bacterial phyla, *Bacteroidetes* and *Firmicutes*, and the obesity-associated microbiota had increased capacity to harvest energy compared to their lean counterpart (113). Of particular importance is a study conducted on twins discordant for body mass, which showed that transplanting gut microbiota to germ-free mice resulted in transmission of obesity, and that it was reversed upon housing with mice transplanted with lean microbiota (114). These studies highlight the significant contribution of gut microbiota towards determining body mass and adiposity.

### 4.1 Shift towards gut dysbiosis in obese state associates with breast cancer progression

**Figure 2:**
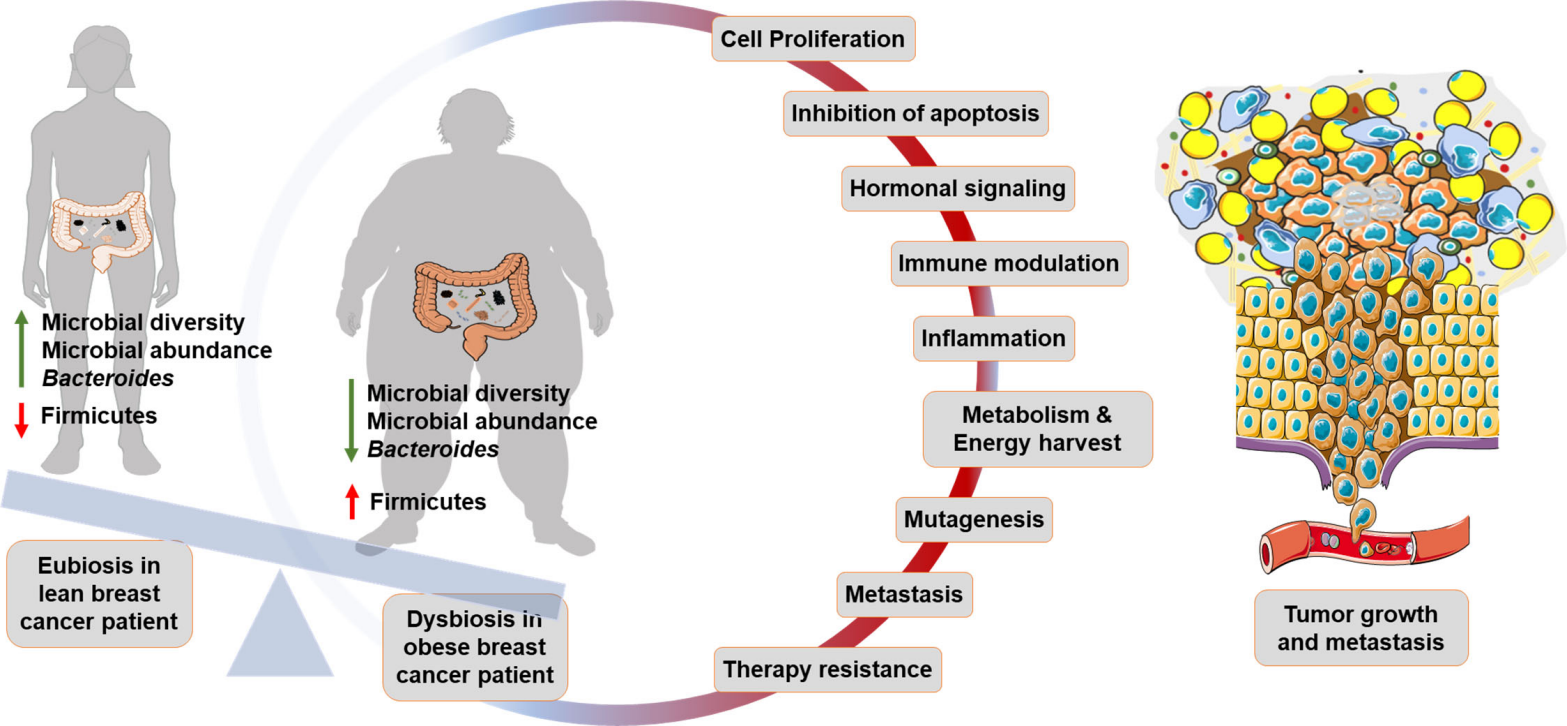
Gut microbiome in obese breast cancer: Lean breast cancer patients generally represent a state of eubiosis within the gut. Dysbiosis in obese breast cancer patients is marked by lower microbial diversity and abundance. Beneficial bacteria such as *Bacteroides* are lower while Firmicutes are higher in obese breast cancer patients. Gut microbial dysbiosis promotes cancer progression by altering physiological processes such as cell proliferation, apoptosis, hormonal signaling, immune modulation, metabolism, induction of mutagenesis, metastasis and therapy resistance.

Gut microbial dysbiosis in obesity is characterized by a less diverse and less abundant flora (10); *Bacteroides*, *Akkermansia muciniphila*, *Faecalibacterium prausnitzii* are typically lower while firmicutes are much higher in obese state (18, 115). Alteration in the host-gut ecosystem disturbs the established physiological homeostasis and may induce carcinogenesis by modulating host cell proliferation, apoptosis, immune cell function, inflammation, hormonal signaling, gene expression and mutagenesis (116–120). For instance, in a recent study conducted on syngeneic breast tumor models in mice, it was noted that gut colonization with the obesity-associated enteropathogenic *Bacteroides fragilis* induced systemic inflammation while reshaping the immune landscape of the tumor and facilitating metastasis to lungs and liver (121). Certain gut bacteria that are modulated in obesity, including *Firmicutes*, *Actinobacteria*, and *Bacteroidetes*, exhibit high levels of β-glucuronidase (β-GUS) or β-glucosidase (β-Gluc) activity (119, 122). These bacteria contribute to the enterohepatic recycling of estrogens by deconjugating them, which raises estrogen levels in the bloodstream and consequently increases breast cancer risk (122, 123). Multiple studies have indicated the existence of modified gut microbiome signatures in breast cancer patients versus matched healthy controls (124–127). A comparison of gut microbiota from 31 breast cancer patients categorized as per BMI (128) showed that the total number of bacteria, and abundance of *Firmicutes*, *Faecalibacterium prausnitzii*, *E. Lenta*, and *Blautia* sp. were lower in overweight and obese breast cancer patients compared to lean breast cancer patients (128), contrary to some prior studies. Overall microbial diversity decreased with higher body fat composition in 32 overweight and obese early-stage breast cancer patients, and an inverse relationship between *A. muciniphila* abundance and body fat was observed (129). Further, the higher BMI and higher ‘Total Body Fat’ (TBF, ≥46%) groups also showed increased prevalence of *Firmicutes* (*f_Clostridiaceae*). Additional observations made from the high TBF group included a higher abundance of *Firmicutes* (*g_Clostridium* and *g_Lachnospira*) and lower abundance of *Actinobacteria* (*f_Coriobacteriaceae*) and *g_Catenibacterium* (130). A similar cross-sectional study on fecal samples from 70 breast cancer survivors indicated a significantly lower relative abundance of *g_Ruminococcus*, *g_Streptococcus*, *g_Roseburia*, and *g_Dorea* in women with a BMI of ≥30 kg/m^2^ (131). The authors also noted an increased abundance of *Proteobacteria* (*g_Proteus* and *g_Pseudomonas*), which are known to be detrimental to gastrointestinal health, in obese breast cancer survivors (132, 133). In addition, the gut microbes, and their metabolites and enzymes regulate metabolism and absorption of orally as well as systemically administered drugs, thereby also affecting response to therapy (11). Studies examining the relationship between gut microbiota and obesity have uncovered interesting microbial connections, where microbes associated with obese state may also fuel breast cancer (**Figure 2**).

### 4.2 Dysbiosis directly and indirectly interferes with cancer therapy

Due to the ability of the host microbial ecosystem to affect various physiological processes, response to cancer therapy is also influenced by gut microbiota composition. This is achieved at multiple levels involving regulation of drug pharmacokinetics such as absorption, metabolism, as well as modulation of drug-induced toxicity and immune responses (11, 134). Gastrointestinal microbes induce biotransformation of drugs through reactions like hydrolysis, reduction, decarboxylation, deconjugation and removal of functional groups, thereby modifying key features that may be necessary for activity (135). Additionally, they affect drug absorption by altering the gut barrier physiology, and also by indirectly regulating expression of hepatic genes involved in xenobiotic metabolism (11, 136). Anticancer-immune response promoting effects of gut microbiota on cyclophosphamide- and platinum- drug based therapies have been studied in mice. Viaud et al. showed that depletion of Gram-positive bacteria in tumor bearing mice caused a reduction in Th17 responses along with resistance to cyclophosphamide (137). Efficacy and inflammatory signatures of platinum chemotherapy on subcutaneous tumors was markedly reduced in germ-free or gut microbiota-depleted mice (138). In addition, several studies have suggested improved antitumor efficacy and reduced toxicity of cisplatin when supplemented with the probiotic bacteria *Lactobacillus acidophilus* (139, 140). Multiple clinical studies on breast cancer patients reveal differences in therapy outcome depending on the microbial diversity in the gut. Interestingly, correlation between gut microbial diversity and response to neoadjuvant chemotherapy in 23 normal range BMI patients with invasive breast cancer (141) showed that the non-responders appeared to have lower abundance and lower diversity of butyrate- and indole-3-propionate- producing bacteria. In general, the non-responders had lower levels of *Firmicutes* while levels of *Bacteroidetes* were higher compared to the responders. Also, differences in T cell infiltration between responders and non-responders were observed, and gut microbial diversity as a prognostic marker to predict neoadjuvant chemotherapy outcome was suggested (141). Fecal metabolite profiling on 8 ER+PR+ breast cancer patients, before and during three cycles of neoadjuvant chemotherapy (142) revealed a significant increase in the levels of butyrate, propionate and acetate after second cycle of the treatment, suggesting that chemotherapy may favor enrichment of gut microbes producing these short chain fatty acids (SCFAs). SCFAs exert physiological effects by inhibiting histone deacetylases (HDACs) and activating G-protein-coupled receptors (GPCRs) such as GPR109A and GPR43 (143, 144). These processes trigger proinflammatory pathways through mechanisms such as suppressing NF-κB activation or inducing lipolysis to release free fatty acids that interact with toll-like receptors (TLRs) Supplementation with butyrate in diet-induced obese mice is also shown to reduce leptin levels, an adipokine that is known to promote tamoxifen resistance (55, 145). Additionally, the good responders had elevated levels of several amino acids compared to poor responders (142). Another study investigated the role of fecal microbiota in determining the efficacy of neoadjuvant trastuzumab-based chemotherapy efficacy in HER2+ breast cancer patients (146). Trastuzumab is a recombinant monoclonal antibody (mAb) that specifically binds to HER2 receptor and represents the first line of treatment for HER2 enriched breast cancer. Gut microbial diversity among 24 patients treated with trastuzumab-based chemotherapy was compared between patients that showed a pathologic complete response vs those that did not (146). The non-responders displayed lower alpha diversity characterized by lower abundance of *Firmicutes f_Lachnospiraceae* and *f_Turicibacteraceae*, *Bacteroidetes f_Prevotellaceae*, *Actinobacteria f_Bifidobacteriaceae*, and *Proteobacteria g_Desulfovibrio*. Additionally, the authors also provided experimental evidence by demonstrating that fecal microbiota transplant from responders to mice resulted in superior Trastuzumab efficacy over those transplanted with fecal microbiota from non-responders, thereby suggesting a potential involvement of the altered microbial population in mediating mAb signaling (146). An *in vitro* study exploring the effects of the SCFA butyrate on HER2 overexpressing SKBR3 breast cancer cell line found that anticancer effects of butyrate were significantly enhanced in combination with trastuzumab via increased p27Kip1 (147). Terrisse et al. reported a favorable transition in gut microbial diversity in early breast cancer patients following chemotherapy (148). While the abundance of microbial signatures associated with healthy volunteers was increased, the over-representation of microbes relevant to poor prognosis was reduced upon chemotherapy treatment. This included reduction in the abundance of *Clostridium* sp, *Bacteroides* sp and *Veillonella* sp. Interestingly, the study also reported a shift in beta diversity toward effectuating neurological side effects and overt weight gain post chemotherapy (148). Involvement of gut microbial signatures with chemotherapy-dependent weight gain was also suggested by another group (149). Fecal microbiota of patients that gained weight following treatment was significantly different in beta diversity from the no-weight gain patients, analyzed before the treatment began (149). Due to the significant impact of gut microbiome in regulating metabolic processes, Juan et al. attempted to use probiotic supplements to counter chemotherapy-induced weight gain (150). Breast cancer patients receiving probiotic supplements containing *Bifidobacterium longum*, *Lactobacillus acidophilus* and *Enterococcus faecalis* displayed smaller differences in docetaxel-induced body weight gain (150). In conclusion, there appears to be an interplay between gut microbiota composition and chemotherapeutic drug action. The ability of microbes to alter not only the efficacy of chemotherapeutic regimens, but also the associated side effects and metabolic changes, warrants in depth investigation. Further characterization of the specific microbial species and processes involved will be crucial in improving therapy outcomes.

## 5 Would obese state associated sustained low-grade inflammation improve response to immunotherapy?

Immune checkpoint blockade (ICB) therapy has revolutionized modern treatment approaches for cancer patients, especially in the case of cancers such as lung cancer and melanoma (151, 152). As the name suggests, ICB utilizes specific mAbs developed against immunosuppressive proteins, such as cytotoxic T-lymphocyte associated protein 4 (CTLA4) or programmed cell death-1 (PD-1) expressed on T cells or programmed cell death ligand-1 (PD-L1) expressed on cancer cells which help them escape the immune surveillance by cytotoxic T cells (152). However, this therapy is not as effective in targeting tumors that are immunologically “cold” characterized by modest immune cell infiltration (151). In case of breast cancers, ICB is only used, either alone or in combination with chemotherapy, in the treatment of advanced TNBC and HER2+ breast cancer cases, where T cell infiltration is markedly higher compared to ER+PR+ luminal A and luminal B subtypes (151, 153). Hence, endocrine therapy and adjuvant chemotherapy continue to be the treatment of choice for early and ER+PR+ breast cancer cases (151, 153). However, cancer in obese state bring in a different set of conditions marked by a chronic inflammatory TME that is rich in immune cells. Consequently, obese cancer patients have shown better response to ICB with improved overall survival (OS) and/or progression free survival (PFS) in some cancers other than breast cancer (7, 154–156). Although clinical studies investigating immunotherapy approaches in breast cancer patients with high BMI are still lacking, insights drawn from preclinical studies and clinical studies in other cancers could prove instrumental in identifying targetable components and improving ICB efficacy. A retrospective investigation encompassing 976 patients diagnosed with melanoma, non-small cell lung cancer (NSCLC), or renal cell carcinoma (RCC), and treated with ICB uncovered that overweight and obese patients displayed a higher response rate in contrast to lean counterparts (157). Kichenadasse et al. also made similar observations in their clinical study focused on 2110 NSCLC patients, where they noted improved overall survival associated with a BMI of ≥ 30 kg/m^2^ following ICB treatment, an effect that was absent in patients undergoing docetaxel chemotherapy (158). Additionally, using mouse tumor models and clinical patient data, Wang et al. demonstrated that obesity led to accelerated immune aging, tumor advancement, and impaired T cell function mediated by PD-1, driven largely by leptin, while also enhancing the effectiveness of PD-1/PD-L1 blockade in both *in vivo* models and cancer patients (155). In essence, obesity exacerbates immune dysfunction and tumor development while simultaneously enhancing ICB efficacy and survival.

## 6 Obese state, gut dysbiosis and immunotherapy efficacy-upcoming evidences and inferences

Growing evidences suggest a role of the gut microbiota in modulating immunotherapy efficacy (159–161). Some of the earliest studies linking intestinal microbiota with immunotherapy were conducted by Sivan et al. and Vetizou et al, who compared tumor growth in germ-free mice upon treatment with anti-PD-L1 and anti-CTLA4, respectively (160, 161). Sivan and colleagues noted improved control of tumor growth in a combination treatment involving anti-PD-L1 therapy and oral administration of the commensal bacteria *Bifidobacterium* (160). Similarly, using antibiotic-treated and germ-free mice tumor models, Vetizou et al. demonstrated significance of *Bacteroides fragilis* specific T cell responses in determining anti-CTLA4 efficacy (161). Other groups analyzing fecal microbiota from melanoma patients, identified enrichment of *Ruminococcaceae* family (24), *Bifidobacterium longum*, *Collinsella aerofaciens*, and *Enterococcus faecium* (25) in patients responsive to anti-PD-1 therapy. The gut microbiome from lung and kidney cancer pateints nonresponsive to PD-1 blockade was found to have lower abundance of *Akkermansia muciniphila* (26). Meanwhile, a few studies have reported unfavorable microbial signatures associated with toxicity resulting from ICB therapy, classified as immune-related adverse events (irAEs) (159, 162). For instance, elevated levels of *Firmicutes* like *Faecalibacterium prausnitzii* were associated with increased incidence of colitis in metastatic melanoma patients treated with Ipilimumab targeting CTLA4 (163). A meta-analysis of anti-PD-L1 treated melanoma patients linked enrichement of *Lachnospiraceae* sp. with favorable clinical response while that of *Streptococcaceae* sp. with unfavorable response and irAEs (164).

### 6.1 Implicating gut microbiota in breast cancer immunotherapy in obese state

Using obese breast cancer preclinical models, Pingili and colleagues demonstrated that although tumors in obese group advanced rapidly, treatment with anti-PD-1 reshaped the local and peripheral TME to increase cytotoxic CD8+ T cell infiltration and proinflammatory M1 macrophages, and decrease immunosuppressive populations like myeloid-derived suppresor cells (MDSCs) (165). The authors also identified the fecal microbiota signature associated with anti-PD-1 treatment marked by an increased abundance of microbes that included *Lactobacillus*, *Bifidobacterium*, *Akkermansia* and *Rikenella*, and reduced abundance of *Bacteroides*, *Paenibacillus*, *Cellulosimicrobium*, and *Enterobacteriaceae* (165). Mechanistic evidence for *Lactobacillus*-mediated improvemnet in anti-PD-1 efficacy was recently reported (166). Using breast, melanoma and colorectal cancer mouse models, the authors showed that *Lactobacillus* strain *L. johnsonii* or its metabolite indole-3-propionic acid (IPA) enhance CD8+ T cell stemness and improve ICB response (166). At the molecular level, IPA induced the formation of progenitor exhausted CD8+ T cells (Tpex) by increasing H3K27 acetylation in the super-enhancer region of Tcf7 that eventually resulted in higher T effector cell numbers thereby boosting anti-PD-1 response. It is also noteworthy that *Bifidobacterium* and *Akkermansia* have been reported to be elevated in other anti-PD-1 trials as well (25, 26). Marge et al. provided mechanistic insights into how *Bifidobacterium* enhances the response to immunotherapy. They demonstrated that *B. pseudolongum* produces inosine, which activates Th1-specific immune responses by binding to the adenosine A_2A_ receptor (A2AR) on T cells. This interaction improved the efficacy of anti-CTLA4 treatment in mouse models of colorectal, melanoma, and bladder cancer (30). The authors also found that *Akkermansia. muciniphila* utilizes a similar inosine-A_2A_R interaction to activate T cells. Beneficial effects of *A.muciniphila* in the management of obesity and metabolic disorders have been well documented (27). *A. muciniphila* has been shown to enhance the anti-PD1 immune checkpoint blockade response in lung and kidney cancers by promoting the recruitment of CCR9+CXCR3+CD4+ T lymphocytes in an interleukin-12-dependent manner (26). Besides, *A. muciniphila* is also known to produce SCFAs such as propionate and acetate that have anti-breast cancer properties (167, 168). Comparing the relative abundance of *Ruminococcus* and *Bacteriodales* in intestinal microbiota from obese mice, Pingili et al. also highlight the importance of *Ruminococcus*/*Bacteroidales* ratio in determining ICB efficacy; a higher abundance of *Ruminococcus* was associated with increased efficacy (165). Some of the bacteria that have been documented to influence T cell function follow common mechanisms. For instance, *Bifidobacterium* and *Akkermansia* produce a metabolite Inosine that binds to adenosine receptors on T cells and induce CD4+ Th1-specific immune responses. Others such as Lactobacillus, appear to rely on the tryptophan metabolite IPA to modulate cytotoxic CD8+ T cell activity. Thus, it appears that the type of response, whether CD4-specific or CD8-specific, is a function of the metabolite that is mediating these effects.

### 6.2 Involvement of gut microbiota in cancer immunotherapy in obese state- learning from other cancers

**Figure 3:**
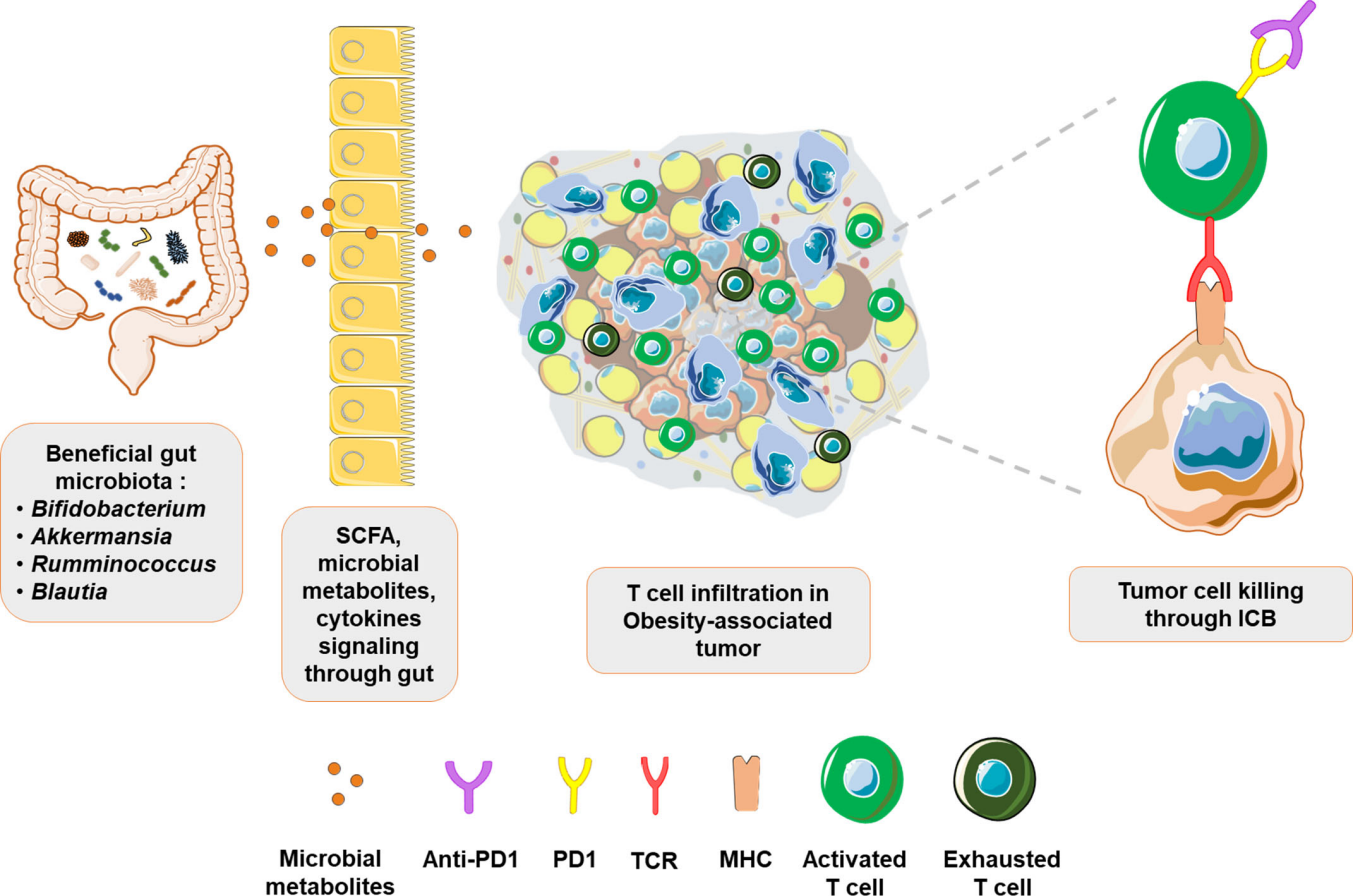
Beneficial gut microbiota improves ICB response in obesity-associated tumors: Evidence suggests that gut microbes such as *Bifidobacterium, Akkermansia, Rumminococcus* and *Blautia* exert beneficial effects through their metabolites. Signaling from the gut improves systemic immune landscape by increasing immune surveillance and immune infiltration in tumors. Consequently, predominantly exhausted T cell phenotype in obesity-associated tumors is transformed into an activated T cells phenotype, with revamped tumor cell targeting and response to immunotherapy.

Fecal microbiota analysis from metastatic melanoma patients showed higher response rates to ICB in *Ruminococcaceae*-enriched patients compared to those enriched for *Bacteroidaceae* (169), corroborating the findings in breast cancer models (165). Thus, there appears to be an inverse corelation between *Ruminococcaceae* and *Bacteroidaceae* distribution in determining disease prognosis. This relationship is perhaps further highlighted in the induction of thermogenesis and alleviation of obesity by *Ruminococcus torques* through the production of deoxycholic acid, a process that is inhibited by *Bacteroides vulgatus* (170). In addition, the abundance of *Ruminococcaceae* family member, *Faecalibacterium* sp. was reduced in a Chinese cohort of breast cancer patients and *F. prausnitzii* inhibited IL6 secretion by MCF7 cells (171). Although a direct metabolite mediating these effects was not identified, it is important to recall IL6 involvement in obesity-associated inflammation (60) and recent findings where IL6 signaling was reported to render resistance to ICB therapy (172). These studies bring *Ruminococcaceae* to the forefront of host-microbiota interactome and call for additional studies focusing on their role in regulating the obesity-breast cancer-ICB therapy interplay. *Blautia* is another interesting gut microbial species that appears to be of importance in obesity and breast cancer. In comparison to lean breast cancer patients, the abundance of *Blautia* sp. is significantly reduced in microbiota of obese breast cancer patients (128). A recent study also identified an inverse relationship between *Blautia* sp. abundance and obesity in gut microbiota of Japanese adults. Moreover, oral administration of *Blautia wexlerae* in mice decreased high fat diet-induced obesity through metabolic reprogramming and anti-inflammatory signaling, effects that were likely mediated through production of succinate, lactate, and acetate (173). It is also noteworthy that abunance of *Blautia* sp. in fecal samples from NSCLC patients undergoing ICB therapy positively correlated with response to therapy along with longer progression free survival (174). Thus, the beneficial effects of *Blautia* in managing obesity and improving ICB efficacy could potentially hold promise for immunotherapy approaches in obese breast cancer patients, warranting further investigation. Extrapolation of observations made in patients, with triple negative breast cancer (TNBC), undergoing immunotherapy could also provide likely targets worth investigating for breast cancer therapy in obese state. For instance, a recent study reported that TNBC tumors in patients responsive to immunotherapy were abundant in *Clostridiales* and their metabolite trimethylamine N-oxide (TMAO) (175). Higher plasma levels of TMAO correlated with active tumor immune microenvironment. Though the source of TMAO in TNBC tumors was speculated to be tumor associated *Clostridiales*, it is noteworthy that HFD-induced obese mice model showed higher *Escherichia coli*-induced choline catabolism leading to elevated circulating levels of TMAO (176). Thus, it will be interesting to investigate choline metabolism and TMAO levels, and their effects on T cell function, in obese breast cancer patients. **Table 1** summarizes gut microbial signatures associated with immunotherapy response and their role in obesity and breast cancer. In conclusion, microbiome-associated interactions with host immune system appear to play vital roles in determining ICB efficacy. Precisely designed clinical studies examining the relevance of this axis in obese breast cancer patients could improve the promise of ICB therapy in obese state, and also provide insights for overall improvement of ICB efficacy (**Figure 3**).

**Table 1:** Gut microbes associated with immunotherapy and their involvement in obesity/breast cancer.

Ref	Study	Family/Genus/Species	Model/Cancer	Therapy	Outcome	Implications/evidence in obesity/obese breast cancer/breast cancer
165	Pingili et al.,	*Akkermansia*	C57B6/J HFD-fed vs LFD-fed. E0771 murine mammary carcinoma	Anti-PD-1	Increased abundance of *Akkermansia*. Lower abundance of *Bacteroides.* Lower tumor burden. Improved immune function.	*A. muciniphila* produced propionate and acetate have anti-breast cancer properties [167, 168]. Beneficial in obesity management [27]
166	Jia et al.,	*Lactobacillus*	C57BL6/J Mc38 mouse colon cancer	Anti-PD-1	Enhanced efficacy with *L. johnsonii* or its metabolite indole-3-propionic acid (IPA) enhances immunotherapy.	IPA supplementation improved anti-PD-1 response and survival in 4T1 and MMTV-PyMT breast cancer mice [166]. Lactobacillus enriched in anti-PD1 therapy responder obese E0771 murine mammary cancer mice [165]
160	Sivan et al.,	*Bifidobacterium*	C57BL/6 - B16 mouse melanoma	Combination treatment {Anti-PD-L1 + oral Bifidobacteria}	Improved efficacy with *Bifidobacteria*	Lower alpha diversity of *Bifidobacteriaceae* in HER2+ breast cancer patients treated with Trastuzumab [146]. • Probiotic supplements containing *Bifidobacterium longum* prevented docetaxel-induced weight gain in breast cancer patients [150]. •*Bifidobacterium* enriched in anti-PD-1 treated obese breast cancer mice [165].
25	Matson et al.,	*Bifidobacterium* *longum*	Metastatic melanoma patients, samples collcted pretreatment	Anti-PD-1	Enriched in responders	
169	Simpson et al.,	*Ruminococcaceae*	Melanoma patients, samples collected pretreatment	Combination treatment {Anti-PD-1+anti-CTLA4} or Anti-PD-1	Enriched in responders	Lower abundance of Ruminococcaceae *g_Faecalibacterium* in breast cancer patients [171]. • Ruminococcus torques produced deoxycholic acid induces thermogenesis, alviates obesity [170]. Improved anti-PD-1 efficacy in Rumminococcus enriched obese breast cancer mice [165].
174	Shijo et al.,	*Blautia* spp	Locally advanced/unresectable or postoperative recurrent NSCLC patients	Anti-PD-1, Anti-PD-L1, either alone or in combination with platinum-based therapy	Enriched in responders	Lower abundance in obese breast cancer patients [128]. • Inverse relationship with obesity in Japanese cohort, oral administration of *B. wexlerae* decreased HFD-induced obesity [173].
175	Wang et al.,	*Clostridiales*	TNBC *tumor samples (not fecal), plasma samples	Anti-PD-1(only patients from whom plasma samples were collected)	*Clostridiales* enriched in tumor samples responders. High plasma levels of trimethylamine N-oxide in anti-PD-1 treated patients.	*Escherichia coli*-induced choline catabolism in HFD-induced obese mice led to elevated circulating levels of trimethylamine N-oxide [176].

## 7 Concluding remarks and future perspectives

Obesity is a major risk factor for developing breast cancer, and a persistent state of chronic low-grade inflammation as well as the presence of immunosuppressive factors that mark obese TME, appears to favor tumor progression. Although immunotherapy interventions in other solid tumors have shown good prognosis in obese cancer patients, such studies are still lacking in breast cancer. In general, ICB has predominantly been effective for the most aggressive TNBC patients. The inflammatory TME and relatively increased immune cell infiltration associated with obese state imparts distinct tumor intrinsic features that may be exploited to improve ICB efficacy in obese breast cancer patients. However, it is also imperative to develop additional strategies to achieve effective immune activation in such tumors. Gut microbiota represents a fairly new and relatively unexplored element of cancer immunotherapy. The past decade has unraveled multitude of host-microbiota interactions that critically regulate physiological processes, including tumor progression at sites physically distant from the gut. Gut microbial dysbiosis in obesity and breast cancer represents a plausible connection that warrants further investigation with meticulously designed preclinical and clinical studies to identify microbiota components with immune-related functions. Insights gained from studies in other cancers will also be vital in designing hypothesis-driven studies to decipher the obesity–breast cancer-microbiome-immunotherapy axis.
